# Structural analysis of the chicken FANCM–MHF complex and its stability

**DOI:** 10.1107/S2053230X20016003

**Published:** 2021-01-01

**Authors:** Sho Ito, Tatsuya Nishino

**Affiliations:** a Department of Biological Science and Technology, Faculty of Industrial Science and Technology, Tokyo University of Science, 6-3-1 Niijyuku, Katsushika-ku, Tokyo 125-8585, Japan

**Keywords:** histone fold, DNA binding, DNA repair, protein complex, X-ray crystallography

## Abstract

FANCM is involved in eukaryotic DNA-damage recognition and activates the Fanconi anemia (FA) pathway through complex formation. MHF is one of the FANCM-associating components and contains a histone-fold DNA-binding domain. Loss of the FANCM–MHF interaction compromises the activation of the FA pathway, resulting in chromosomal instability. Thus, formation of the FANCM–MHF complex is important for function, but its nature largely remains elusive. Here, the aim was to reveal the molecular and structural basis for the stability of the FANCM–MHF complex. A recombinant tripartite complex containing chicken FANCM (MHF interaction region), MHF1 and MHF2 was expressed and purified. The purified tripartite complex was crystallized under various conditions and three different crystals were obtained from similar crystallization conditions. Unexpectedly, structure determination revealed that one of the crystals contained the FANCM–MHF complex but that the other two contained the MHF complex without FANCM. How FANCM dissociates from MHF was further investigated and it was found that the presence of 2-methyl-2,4-pentanediol (MPD) and an oxidative environment may have promoted its release. However, under these conditions MHF retained its complexed form. FANCM–MHF interaction involves a mixture of hydrophobic/hydrophilic interactions, and chicken FANCM contains several nonconserved cysteines within this region which may lead to aggregation with other FANCM–MHF molecules. These results indicate an unexpected nature of the FANCM–MHF complex and the data can be used to improve the stability of the complex for biochemical and structural analyses.

## Abstract 2

Three crystals of MHF and its complex with FANCM were obtained, and structural analysis revealed not only their structures but the unexpected release of FANCM from MHF. The biochemical stability of the FANCM–MHF complex was analyzed and it was found that an oxidative environment and organic solvent promote the aggregation and dissociation of the complex.

## 1. Introduction   

FANCM is a component of the Fanconi anemia DNA-repair system that eliminates inter-strand cross-links (Milletti *et al.*, 2020 ▸). It consists of an N-terminal helicase domain that exhibits homology to the SF2 helicase, and a C-terminal nuclease-like domain that is related to other heterodimeric endonucleases but lacks catalytic residues (Meetei *et al.*, 2005 ▸; Whitby, 2010 ▸; Xue *et al.*, 2015 ▸). In addition to these two terminal domains, FANCM has several conserved sequence motifs for protein–protein interaction (Deans & West, 2009 ▸). The FANCM-associated histone-fold protein (MHF) 1/2 complex (also known as CENP-S/CENP-X) associates with the conserved region of FANCM immediately after the helicase domain to form a FANCM–MHF complex (Singh *et al.*, 2010 ▸; Yan *et al.*, 2010 ▸). This complex is conserved from yeast to humans (Blackford *et al.*, 2012 ▸; Singh *et al.*, 2010 ▸) and is thought to play key roles in repair processes. MHF stabilizes FANCM and promotes its DNA-binding activity (Singh *et al.*, 2010 ▸; Yan *et al.*, 2010 ▸). Mutations affecting its DNA binding or FANCM interaction compromise the stability of FANCM and the recruitment of other components to DNA damage sites (Fox *et al.*, 2014 ▸; Singh *et al.*, 2010 ▸; Tao *et al.*, 2012 ▸; Yan *et al.*, 2010 ▸). Thus, FANCM–MHF complex formation is important for the DNA damage-response pathway. Several structures of human FANCM–MHF complexes are known, and they form heteropentamers (Fox *et al.*, 2014 ▸; Tao *et al.*, 2012 ▸). FANCM binds asymmetrically to MHF mainly through an extensive surface area containing hydrogen bonds, salt bridges and hydrophobic interactions. The large surface area between FANCM and MHF suggests that the complex is relatively stable, but its exact nature remains elusive.

Here, using the chicken FANCM–MHF complex, we tried to elucidate the stability of the complex through structural and functional analyses. The sequence conservation of the MHF-interacting region of FANCM between chicken and human is less than 50%. We purified the complex and obtained crystals. Surprisingly, we found that several crystals only contained MHF, whereas others retained the FANCM–MHF complex. We found conditions that promoted the release of FANCM from MHF, which may be important for biochemical and structural analysis.

## 2. Methods   

### 2.1. Protein expression   

The chicken FANCM gene was cloned from the chicken DT40 cDNA library. A recombinant plasmid encoding MBP-6×His-TEV protease-recognition site (tev)-FANCM (amino acids 660–804), 6×His-tev-MHF1 (truncated at residue Asn104) and StrepII-tev-MHF2 was used to transform *Escherichia coli* cells [BL21 Star (Thermo Fisher) with the pRARE2LysS plasmid (Novagen)]. Transformed cells were grown in LB or Terrific Broth containing 1 m*M* ampicillin at 37°C until the OD_600_ reached 0.7–1.0. Protein expression was induced by the addition of 0.2 m*M* isopropyl β-d-1-thio­galactopyranoside and the culture was incubated at 20°C for 12–15 h. The cells were harvested by centrifugation using a JLA8.1000A rotor (Beckman) at 4000 rev min^−1^ for 15 min at 4°C and the bacterial pellet was stored at −80°C until purification.

### 2.2. Protein purification   

The FANCM–MHF bacterial pellet was thawed and resuspended in buffer consisting of 10 m*M* Tris–HCl pH 8.0, 500 m*M* NaCl, 0.06% Polyethyleneimine P70 (Wako). The resuspended cells were sonicated using a Misonix Sonicator XL2020 ultrasonic homogenizer. The lysate was centrifuged using an R18A rotor (Beckman) at 11 500 rev min^−1^ for 15 min at 4°C. The supernatant was applied onto a 30 ml HisTrap FF crude column (GE Healthcare) and the bound proteins were eluted using 500 m*M* imidazole. The eluate was concentrated to 2 or 5 ml by ultrafiltration with Amicon Ultra centrifugal filters (molecular-weight cutoff 50 000; Millipore) at 5000*g* and 4°C and loaded onto a Superdex 200 pg column (GE Healthcare). Peak fractions were cleaved by the addition of homemade TEV protease and 1 m*M* dithiothreitol (DTT) at room temperature overnight. The solution was applied onto a HisTrap FF crude column. The flowthrough fractions were concentrated by ultrafiltration with Amicon Ultra centrifugal filters (molecular-weight cutoff 50 000) and loaded onto a Superdex 200 pg column. The peak fractions containing FANCM, MHF1 and MHF2 were concentrated by ultrafiltration with Amicon Ultra centrifugal filters (molecular-weight cutoff 50 000) and stored at −25°C. All columns were equilibrated with 10 m*M* Tris–HCl pH 8.0, 500 m*M* NaCl.

The quality of the protein purification was analyzed by SDS–PAGE using a 15% gel. The protein concentration was measured by UV absorption at 280 nm and was calculated using an extinction coefficient ɛ = 32 680 and a molecular weight of 59 683 for heteropentameric FANCM–MHF.

### 2.3. Crystallization and cryopreservation   

Initial crystallization was carried out using a Mosquito crystallization robot (TTP Labtech). FANCM–MHF was crystallized by mixing 100 nl protein solution and 100 nl Morpheus MD-HT screen buffer G12 [0.1 *M* Tris–bicine pH 8.5, 0.1 *M* carboxylic acids mix, 12.5% 2-methyl-2,4-pentanediol (MPD), 12.5% PEG 1000, 12.5% PEG 3350; Molecular Dimensions]. Needle-shaped and tetrahedral crystals appeared after seven days. The crystals were soaked in the crystallization buffer for diffraction measurements. Manual crystallization was carried out by sitting-drop crystallization at 20°C using 1 µl concentrated protein solution and 1 µl homemade crystallization reagent (0.1 *M* MOPS–HEPES pH 7.0, 0.1 *M* carboxylic acids mix, 15% MPD, 20% PEG 3350, 300 m*M* NaCl). Crystals appeared after 2–10 days. The crystals were transferred to and soaked in 0.1 *M* MOPS–HEPES pH 7.0, 15% MPD, 25% PEG 3350, 350 m*M* NaCl supplemented with 0.6 *M* 1,6-hexanediol for 2 h.

### 2.4. Data collection and analysis   

Diffraction data for FANCM–MHF were collected on BL1A and BL17A at Photon Factory and BL44XU at Spring-8. Data were processed with the *HKL*-2000 package (HKL Research). Molecular replacement was carried out using *Phaser* in the *Phenix* suite (Liebschner *et al.*, 2019 ▸). Refinement was also carried out using *Phenix* in refinement mode and model building using *Coot* (Emsley *et al.*, 2010 ▸). Data-collection and final refinement statistics are summarized in Table 1 ▸. The structures were visualized with *Discovery Studio* (Biovia).

### 2.5. Native PAGE analysis   

Protein solution containing 5 µl 2 mg ml^−1^ FANCM–MHF and 5 µl A0 buffer (10 m*M* Tris–HCl pH 8), 10–60% MPD or a mixture of ten carboxylic acids was incubated at 20°C for 60 min and 2 µl native PAGE loading buffer (0.25 *M* Tris–HCl pH 6.5, 20% sucrose, 0.02% BPB) was then added. 10 µl of the mixture was loaded onto 6% polyacrylamide TBE (Tris, boric acid, EDTA pH 8.0) gel. The gel was run at 150 V for 75–90 min in 0.5× TBE buffer at 4°C and subsequently stained with Coomassie Brilliant Blue R-250. The bands that migrated on the TBE gel were clipped out, mashed, suspended in 1× SDS–PAGE loading buffer (0.05 *M* Tris–HCl pH 6.5, 4% sucrose, 0.005% BPB, 5% β-mercaptoethanol, 2% SDS) and incubated at 94°C for 5 min. The supernatant was loaded onto an SDS–PAGE gel. The band intensities were quantified with *ImageJ*.

## 3. Results and discussion   

**Figure 1:**
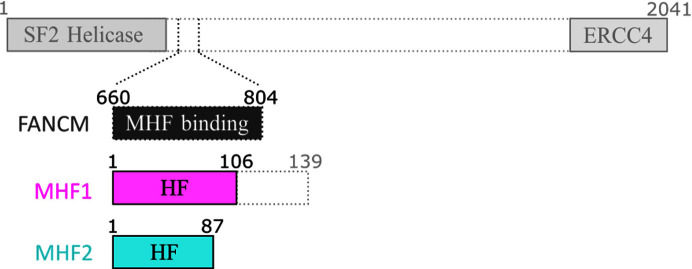
Schematic domain structures of chicken FANCM, MHF1 and MHF2. FANCM has an SF2 helicase domain at the N-terminus followed by an MHF interaction domain and an ERCC4 nuclease-like domain at the C-­terminus. MHF binding indicates an interface with MHF. MHF is composed of MHF1 and MHF2, both of which contain a histone fold (HF). MHF1 contains an additional intrinsically disordered region. For crystallization and biochemical analysis, MHF1 was truncated at Glu106.

**Figure 2:**
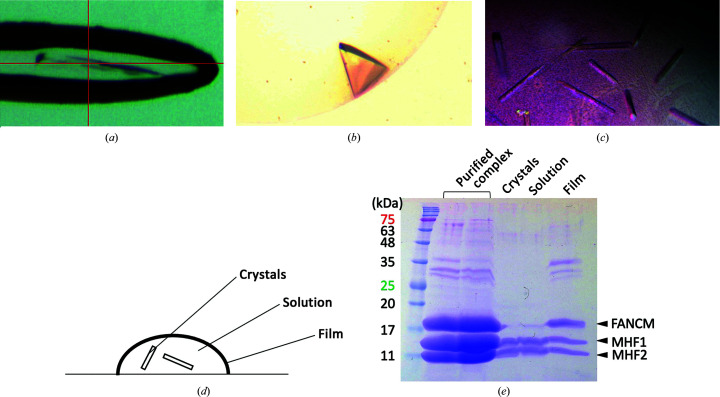
Crystals of FANCM–MHF and its analysis. (*a*–*c*) Photographs of (*a*) a needle-shaped crystal, (*b*) a tetrahedral crystal and (*c*) rod-shaped crystals. (*d*, *e*) Analysis of the crystal drop from the crystallization condition for the rod-shaped crystal. (*d*) Schematic drawing of the sitting-drop crystallization setup indicating crystals, the solution and the film. (*e*) Analysis of each component by 15% SDS–PAGE. The gel was stained with Coomassie Brilliant Blue.

We co-expressed and purified recombinant chicken FANCM–MHF complex consisting of a region of chicken FANCM that interacts with MHF (referred to as FANCM; amino acids 660–804), MHF1 with a C-terminal truncation, and MHF2 (Fig. 1 ▸). The purified FANCM–MHF complex was subjected to crystallization trials. From the initial screening using a crystallization robot, we obtained two different crystal forms (needle-shaped, Fig. 2 ▸ *a*, and tetrahedral, Fig. 2 ▸ *b*) from the same crystallization condition. We then manually refined the crystallization condition. However, the crystallization drop contained heavy oil droplets and clusters of needle-shaped crystals. To improve the quality of the crystals so that they were suitable for diffraction experiments, we optimized the crystallization condition and obtained rod-shaped crystals (Fig. 2 ▸ *c*). In the crystal droplet, a heavy film-like structure formed at the air–liquid interface (Fig. 2 ▸ *d*). We found that the film contained FANCM and MHF, whereas the crystal and the solution consisted mostly of MHF and only a small fraction of FANCM was detected (Fig. 2 ▸ *e*). We could not detect the isolated FANCM protein that was released from the FANCM–MHF complex. The addition of reducing agents such as dithiothreitol partially reduced film formation.

**Figure 3:**
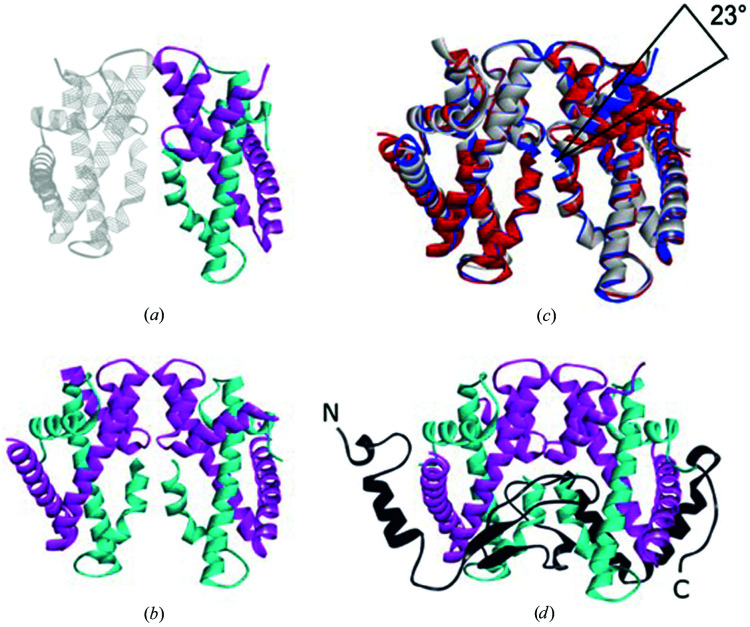
Crystal structures of MHF and FANCM–MHF. (*a*, *b*, *d*) Crystal structures of (*a*) MHF from the crystal in Fig. 2 ▸(*b*), (*b*) MHF from the crystals in Fig. 2 ▸(*c*) and (*d*) FANCM–MHF from the crystal in Fig. 2 ▸(*a*). MHF1, MHF2 and FANCM are colored cyan, magenta and black, respectively. The crystallographic symmetry heterodimer is colored in transparent mode in (*a*). N and C indicate the N- and C-termini of FANCM in (*d*). (*c*) Superimposition of three MHF tetramers from (*a*) in blue, (*b*) in red and PDB entry 3b0b in gray. One of the MHF1 α4 helices in (*b*) deviates from the others by 23°.

Next, we solved the crystal structures by molecular replacement using the previously determined crystal structure of MHF (PDB entry 3b0b; Nishino *et al.*, 2012 ▸). The tetrahedral and rod-shaped crystals contained only MHF. The tetrahedral crystal belonged to space group *C*2 and the structure was refined to 1.25 Å resolution (PDB entry 7da0). It contained one heterodimer in the asymmetric unit and formed a heterotetramer through crystal symmetry (Fig. 3 ▸ *a*). Owing to its high resolution, 169 residues and 249 solvent molecules were placed. The rod-shaped crystal belonged to space group *P*4_1_2_1_2 and the structure was refined to 2.0 Å resolution (PDB entry 7da1). It contained one heterotetramer in the asymmetric unit (Fig. 3 ▸ *b*). The MHF structures from the tetrahedral and the rod-shaped crystals and the previously determined structure (PDB entry 3b0b; Nishino *et al.*, 2012 ▸) were highly similar to each other. The root-mean-square displacement (r.m.s.d.) of the heterodimer region was 0.5–0.6 Å. The structural alignment of the heterotetramer also showed high similarity (r.m.s.d. of 0.8 Å). One noticeable difference was that the orientation of the MHF1 α4 helix in the rod-shaped crystal deviated from the other structures by making a 23° rotation, which was possibly induced by the crystal packing (Fig. 3 ▸ *c*).

On the other hand, structure determination of the needle-shaped crystal indicated that it contained the FANCM–MHF complex (Fig. 3 ▸ *d*). The crystal belonged to space group *P*2_1_2_1_2_1_ and the structure was refined to 2.8 Å resolution (PDB entry 7da2). It contained one FANCM–MHF heteropentamer in the asymmetric unit, with FANCM asymmetrically bound to the MHF heterotetramer. We could model most of MHF and FANCM except for the N-terminal 13 residues. The chicken structure was similar to that of the human complex, with r.m.s.d.s of 2.0 Å (PDB entry 4e45; Fox *et al.*, 2014 ▸) and 2.2 Å (PDB entry 4drb; Tao *et al.*, 2012 ▸). In human FANCM, several flexible loops were not resolved in the crystal structure. Nevertheless, the secondary structure of chicken FANCM was found in similar regions. In summary, we obtained the structure of the FANCM–MHF complex and two different MHF structures from the purified tripartite complex.

**Figure 4:**
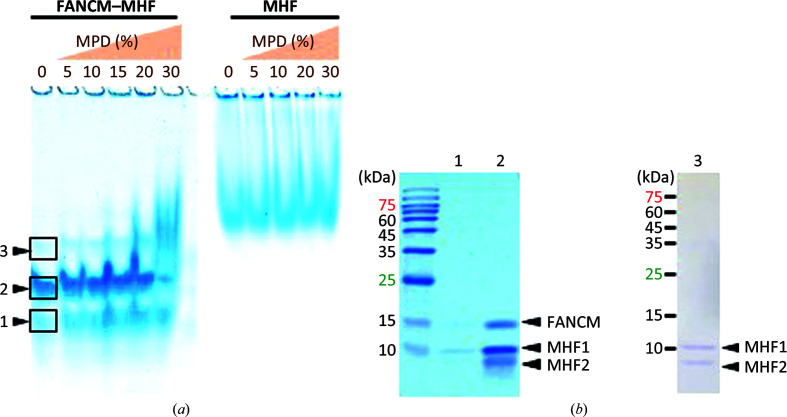
Native PAGE and SDS–PAGE analysis of the FANCM–MHF and MHF complexes in the presence of MPD. (*a*) FANCM–MHF (left) and MHF (right) were mixed with different concentrations of MPD and separated on native PAGE. (*b*) The boxed regions containing the Coomassie-stained bands 1–3 in (*a*) were sliced and separated by 15% SDS–PAGE.

It was surprising that the FANCM–MHF complex disassembled and MHF formed crystals on its own. This suggests that a small fraction of MHF was present in the purified tripartite complex or that components in the crystallization condition may have disrupted the complex. To further assess the nature and stability of the FANCM–MHF complex, we performed native PAGE (Fig. 4 ▸ *a*). Comparison of FANCM–MHF (left) and MHF (right) indicates that both complexes migrate as a discrete band and that the former complex migrates faster than the latter. We confirmed the content by cutting out the bands and analyzing them by SDS–PAGE (Fig. 4 ▸ *b*). The purified FANCM–MHF seems to be nearly homogenous as judged by the gel-filtration peak (Supplementary Fig. S1) and the major fraction was indeed FANCM–MHF (lanes 1 and 2 in Fig. 4 ▸ *b*); however, there was a small amount (∼3%) of MHF without FANCM (lane 3 in Fig. 4 ▸ *b*). Interestingly, when we added 2-methyl-2,4-pentanediol (MPD) the band pattern did not change until 20% MPD, but a further increase to 30% MPD resulted in the formation of a smeared band, which migrated at a position similar to MHF. MHF did not form such a band even at 30% MPD, which suggests that MPD promotes the dissociation of FANCM from the complex. Other components in the crystallization buffer such as a carboxylic acid mixture did not cause such an effect (data not shown). As the film formed during crystallization, and was likely to be caused by the exposure to the air, we speculate that oxidation and MPD promote distortion and dissociation of the complex.

**Figure 5:**
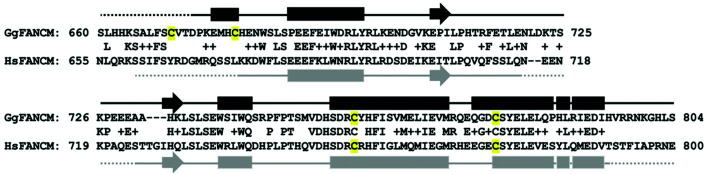
Sequence and secondary-structure alignment of chicken and human FANCM. Chicken FANCM (GgFANCM) and human FANCM (HsFANCM) were aligned by *BLAST* pairwise sequence alignment. The numbers indicate amino-acid residues in each species. The letters between the two sequences indicate identical residues; similar residues are marked ‘+’. Secondary structures of chicken and human FANCM are colored black and gray, respectively. Rectangles, arrows and solid lines represent α-helix, β-sheet and random-coil regions, respectively. Dotted lines are disordered regions. Cysteine residues are highlighted in yellow.

**Figure 6:**
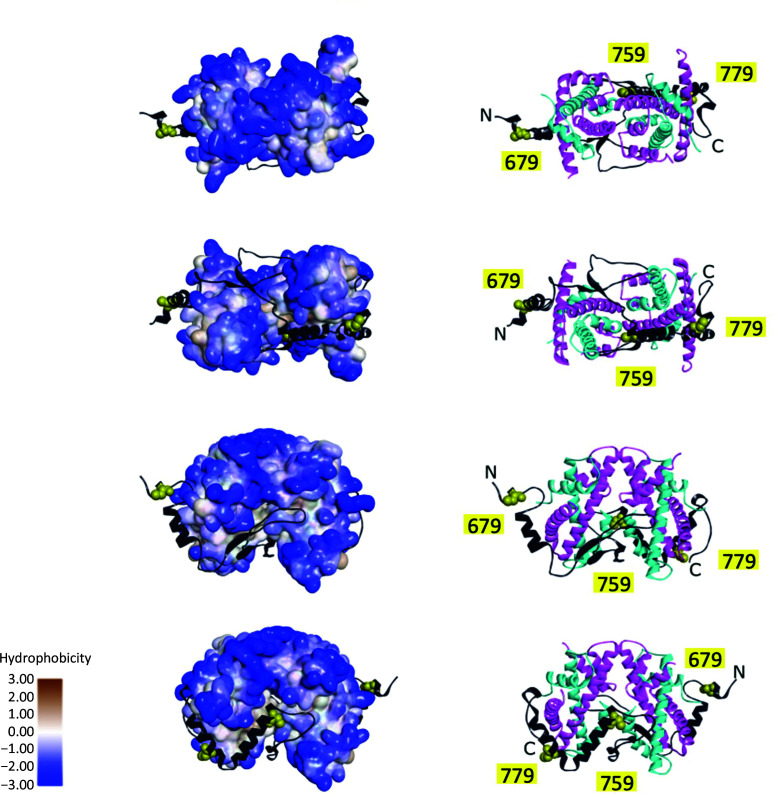
FANCM is embedded in hydrophobic regions of MHF. Ribbon diagrams and protein surfaces of the chicken FANCM–MHF complex are displayed using *Discovery Studio*. The hydrophobicity of each view of the protein surface is displayed (brown, hydrophobic; blue, hydrophilic). N and C indicate the N- and C-termini of FANCM, respectively. The hydrophobic area of MHF is covered with bound FANCM. Cysteine residues are numbered, displayed using CPK style and colored yellow.

What could the explanation be for the dissociation of FANCM from MHF? Sequence analysis of chicken FANCM indicates that there are four cysteine residues within the current construct and two of them are conserved between chicken and human FANCM (Fig. 5 ▸). In the human FANCM–MHF structure (PDB entry 4e45; Fox *et al.*, 2014 ▸), one of the cysteine residues coordinates a zinc ion. In the current chicken FANCM–MHF crystal structure, the corresponding cysteine residue (Cys759) does not coordinate an ion. Within this region, the side chains of Gln76 and Asp80 of MHF2 were present and no isolated electron density for water or ions was found. This coordination is similar to another human FANCM–MHF structure (PDB entry 4drb; Tao *et al.*, 2012 ▸). Cys759 is buried within the complex and may not be sensitive to oxidation (Fig. 6 ▸). On the other hand, Cys670, Cys679 and Cys779 are exposed to solvent; in particular, Cys670 is in the disordered region and may be sensitive to oxidation. Cys679 and Cys779 are located within the α-helix and may be less sensitive. Thus, the truncation of the disordered region in the current chicken FANCM construct might increase the stability of the complex. As for the effect of MPD, it is a commonly used organic solvent in crystallography owing to its amphiphilic nature and small flexible structure that binds to many parts of the protein. FANCM interacts with MHF through an extensive surface area of more than 5000 Å^2^. The interaction is formed by hydrogen bonding, salt bridges and hydrophobic interactions. Mapping of MHF hydrophobicity indicates that the interface is more hydrophobic than the other regions (Fig. 6 ▸). Therefore, MPD might bind and promote the release of FANCM. The released FANCM on its own is unstable and is prone to aggregation, and binds to other FANCM–MHF complexes through its hydrophobic surface and cysteine disulfide bridges. Taken together, our structural analysis revealed the structure of chicken FANCM–MHF and unexpected MHF structures. Biochemical analysis suggests that oxidation and hydrophobic interactions play roles in the stability of the complex. These data could be used to improve the construct for future biochemical and structural analyses.

## Supplementary Material

Supplementary Figure S1. DOI: 10.1107/S2053230X20016003/nw5107sup1.pdf

PDB reference: MHF, 7da0

PDB reference: 7da1

PDB reference: FANCM–MHF, 7da2

## Figures and Tables

**Table 1:** Data-collection and refinement statistics Values in parentheses are for the highest resolution shell.

	MHF (tetrahedral crystal)	MHF (rod-shaped crystal)	FANCM–MHF
PDB code	7da0	7da1	7da2
Wavelength (Å)	0.9	1.1	0.98
Resolution range (Å)	34.01–1.25 (1.295–1.25)	36.5–2.01 (2.082–2.01)	45.17–2.79 (2.89–2.79)
Space group	*C*121	*P*4_1_2_1_2	*P*2_1_2_1_2_1_
*a*, *b*, *c* (Å)	50.566, 69.071, 48.878	59.432, 59.432, 220.949	71.249, 78.469, 87.468
α, β, γ (°)	90, 103.666, 90	90, 90, 90	90, 90, 90
Total reflections	160822	347250	76771
Unique reflections	43638 (4340)	27443 (2661)	12641 (1232)
Multiplicity	3.7	12.6	6.0
Completeness (%)	96.74 (96.21)	99.85 (99.81)	99.32 (99.84)
Mean *I*/σ(*I*)	36.64 (2.17)	22.3 (3.00)	32.0 (4.32)
Wilson *B* factor (Å^2^)	15.40	24.66	55.02
*R*_merge_	0.056 (0.851)	0.103 (0.772)	0.133 (0.788)
*R*_meas_	0.066 (0.999)	0.108 (0.802)	0.145 (0.873)
*R*_p.i.m._	0.034 (0.520)	0.030 (0.217)	0.059 (0.370)
CC_1/2_ †	NA (0.407)	NA (0.930)	NA (0.427)
CC*†	NA (0.761)	NA (0.982)	NA (0.774)
Reflections used in refinement	43628 (4337)	27434 (2662)	12622 (1232)
Reflections used for *R*_free_	2000 (199)	1988 (193)	632 (63)
*R*_work_	0.1927 (0.2966)	0.1968 (0.2075)	0.2161 (0.2829)
*R*_free_	0.2124 (0.2958)	0.2244 (0.2563)	0.2616 (0.3522)
No. of non-H atoms			
Total	1619	2878	3858
Macromolecules	1358	2704	3811
Solvent	249	174	47
No. of protein residues	169	338	469
R.m.s.d., bonds (Å)	0.009	0.007	0.003
R.m.s.d., angles (°)	0.98	0.91	0.53
Ramachandran favored (%)	100	99.7	94.77
Ramachandran allowed (%)	0	0.3	4.36
Ramachandran outliers (%)	0	0	0.87
Rotamer outliers (%)	2.11	0	3.43
Clashscore	5.44	5.82	7.85
Average *B* factor (Å^2^)			
Overall	24.88	31.39	66.05
Macromolecules	22.73	31.03	66.02
Solvent	36.02	36.91	68.26
No. of TLS groups	6	15	20

† Average CC_1/2_ and CC* values were not reported by the version of *HKL*-2000/*SCALEPACK*.
