# Supplementary material for: Structural analysis of the chicken FANCM–MHF complex and its stability
Source: Acta Crystallogr F Struct Biol Commun. 2021 Jan 1;77(Pt 1):1–7. doi: 10.1107/S2053230X20016003 (PMC7805551; doi:10.1107/S2053230X20016003)
Supplement: Supplementary file 1 [file f-77-00001-sup1.pdf]

STRUCTURAL BIOLOGY
COMMUNICATIONS

Volume 77 (2021)

Supporting information for article:

Structural analysis of the chicken FANCM–MHF complex and its stability

Sho Ito and Tatsuya Nishino

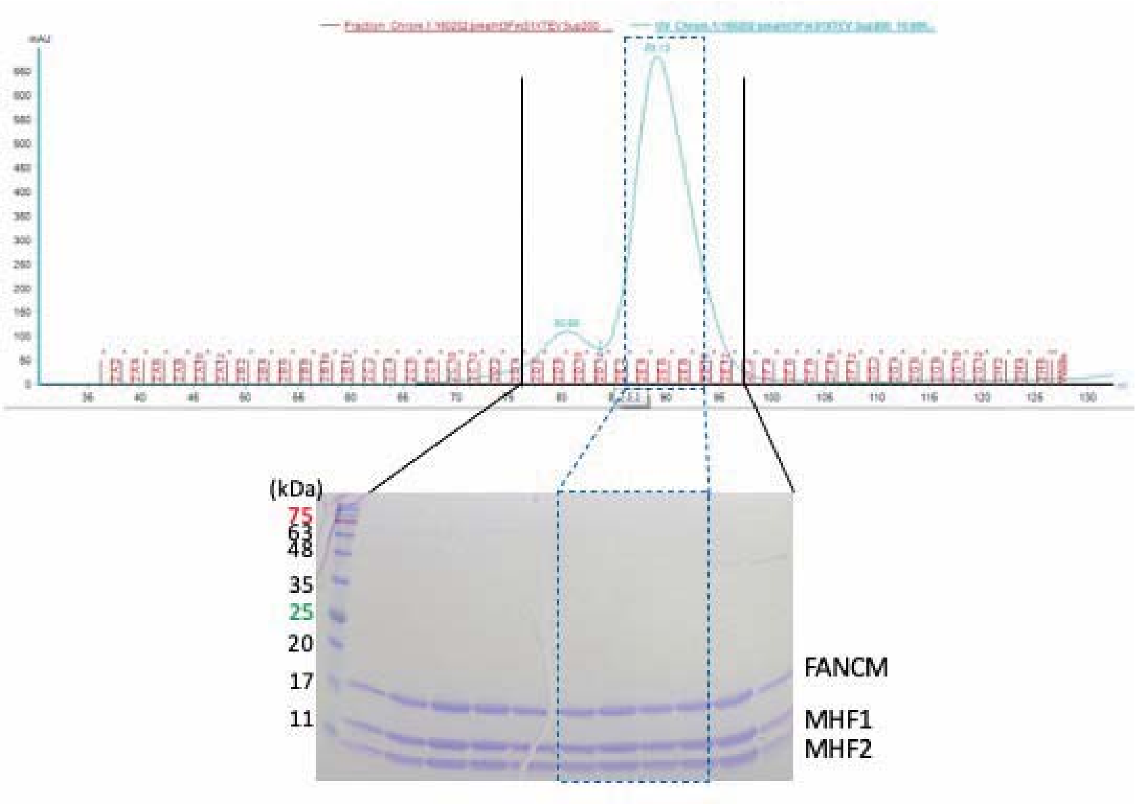

Figure S1 Gel filtration profile and SDS-PAGE analysis of purified FANCM-MHF complex.

Fractions surrounded by blue dashed lines were collected and concentrated by ultrafiltration.
